# Heterogeneity of the Level of Activity of Lgr5+ Intestinal Stem Cells

**Published:** 2014

**Authors:** Shirin Moossavi

**Affiliations:** Digestive Oncology Research Center, Digestive Disease Research Institute, Tehran University of Medical Sciences, Tehran, Iran.

**Keywords:** Intestinal stem cells, heterogeneity, dormancy

## Abstract

Intestinal stem cells (ISCs) are a group of rare cells located in the intestinal crypts which are responsible for the maintenance of the intestinal epithelial homeostasis and regeneration following injury or inflammation. Lineage tracing experiments in mice have proven that ISCs can repopulate the entire intestinal crypt. It is noteworthy that in such experiments, only a subset of intestinal crypts is marked by the specific marker. This is suggestive of different levels of activity of stem cells in different crypts i.e*. *intracryptal variation. Niche succession i.e*.* dominating the entire crypt by the progenies of one stem cell is also suggestive of the intercryptal stem cell heterogeneity. Regional differences in crypt size, proliferative index, and distribution of proliferative cells along the crypt axis have been reported. It is conceivable that ISCs are heterogeneous in terms of their levels of activity. Appreciation of such heterogeneity will significantly challenge the way in which ISCs are investigated. A better understanding of ISC biology will in turn improve our mechanistic understanding of major intestinal disease including inflammatory bowel disease and colorectal cancer.

The intestinal epithelial layer is organized into distinct anatomical features called villi which are protrusions in the lumen, and crypts of Lieberkühn which are invaginations of the epithelial layer into the lamina propria. Villi are absent in the colon. Intestinal stem cells (ISCs) are a group of rare cells located in the intestinal crypts which are responsible for the maintenance of the intestinal epithelial homeostasis and regeneration following injury. Stem cells are quiescent cells with infrequent proliferation; the result of which is usually a new stem cell and a transient-amplifying cell (TAC). TACs differentiate into intestinal epithelial subtypes, i.e. enterocytes, goblet cells, neuroendocrine cells, and Paneth cells; which migrate upward along the crypt axis. Paneth cells are an exception to the migration pattern of mature epithelial cells as they reside at the base of the crypts. It is noteworthy that Paneth cells do not exist in the colon under physiologic conditions but appear in the chronically inflamed colon. ISCs are believed to reside in a functionally defined ‘niche’ at the base of the crypt. The stem cell niche maintains the stem cell properties and regulates the quiescence /proliferation of the stem cell via stem cell regulatory pathways namely Wnt, Notch, BMP and Hedgehog pathways (1). ISCs are defined by their self-renewal capacity and multipotency. Multiple stem cells exist at the base of each crypt. However, only a single stem cell dominates the niche (niche succession); followed by cellular domination of the crypt (monoclonal conversion) (2, 3). This is suggestive of a level of heterogeneity in ISC activity. Our understanding of the ISCs has extensively expanded over the past few decades. It is already known that two types of fast-and slow-cycling stem cells reside in the lower crypts; which are different in terms of cell cycle activity (4). However, it is less clear whether the members of each group also have distinctive characteristics. Hereby, the evidence for the heterogeneity of Lgr5+ ISCs is summarized.

**Intestinal stem cells**

In the murine small intestine, two positions are suggested for the stem cells: at the base of the crypt and at the position 4 from the base of the crypt. The crypt base columnar (CBC) cells reside at the base of the crypt interspersed by the Paneth cells. They are positive for Ki67 proliferation marker and uptake BrdU as early as 6 hours post injection. Their cycling rate is estimated to be once every 24 hours. The CBC cells are Lgr 5 positive and are rapid cycling (5). The position 4 cells, on the other hand, are thought to be quiescent and slow cycling. They are capable of label-retention and are located at position 2-7 immediately above the Paneth cells. Slow cycling cells have been historically identified by their ability to retain BrdU which is diluted in the cycling cells of the crypt. Recently, they have been found to express Bmi1 and mTert (6, 7). Position 4 stem cells do not exist in the colon and their role as ISCs are controversial. For example, label-retaining position 4 cells have been observed to be secretory cell precursors (8). On the other hand, markers of the position 4 cells namely Bmi1, Tert, Lrig1, and Hopx have been found to be either expressed in the Lgr5+ cells or uniformly along the crypt axis (9, 10). It has recently been demonstrated that Lgr5 and Bmi1 mark non- overlapping stem cell populations in mice. CBCs and position 4 stem cells seem to demonstrate distinct functional properties. In a systematic comparison of the two cell population in the murine small intestine, it was found that under steady state, Bmi1^+^cells are more quiescent and less efficiently contribute to the epithelial turnover compared to Lgr5^+^ISCs. In addition, modulation of the Wnt pathway by intravenous adenoviral delivery of either Wnt agonist, R-Spondin1 (Rspo1), or antagonist, Dickkopf-1 (Dkk1), does not affect Bmi1^+^ cells whilst Lgr5^+^cells are highly sensitive to the level of Wnt pathway activity. Lgr5^+^cells are completely lost after 12 Gy whole body γ-irradiation and reappear at a very low frequency 4.5 to 7 days post-irradiation. In contrast, Bmi1^+^ cells are radioresistant and do not quantitatively change. However, they demonstrate a significant increase in the proliferation rate and niche succession post-irradiation. It is suggested that Lgr5^+^cells are the active ISCs under homeostatic condition whereas Bmi1^+^cells are responsible for epithelial regeneration upon injury (4). In an attempt to investigate the consequence of loss of Lgr5^+^CBCs, Tian et al. knocked in diphtheria toxin receptor in the Lgr5 locus. Upon long-term administration of the toxin, CBCs are ablated. However, the crypt anatomy is preserved. CBC-deficient crypts are also capable of generating organoids *in vitro* with comparable efficiency to the control. Upon removal of the toxin from the medium, Lgr5 expressing cells reappear in the organoids. In other words, the regenerational capacity of the crypt is preserved in spite of loss of Lgr5^+^ISCs. Hence, Bmi1^+^ cells are suggested to be the quiescent ISCs which function upon injury. It was observed that Bmi1^+^cells are expanded in Lgr5^+^cells-depleted crypts in the proximal small intestine. It is concluded that Bmi1^+^cells function as reserve stem cells upon damage or loss of more rapidly cycling Lgr5^+ ^cells (11).

**Intestinal stem cell heterogeneity**

Stem cell heterogeneity has been described for embryonic (12-16), muscle (17), hematopoietic (18, 19), neural (20), and induced pluripotent stem cells (21). Stem cell heterogeneity at the level of dormancy has been described in murine hair follicle. The hair follicle undergoes cycling phases of destruction, rest and rapid proliferation which are regulated by major signalling pathways including Wnt, TGF-β, and BMP. Janich et al. demonstrated that the bulge CD34^+^/α6 integrin^high^ stem cells have distinct levels of clock pathway activity at each stage of the hair cycling. Different state of the clock activity was further shown to affect the stem cell regulatory pathways such as Wnt and TGF-β at gene expression level (22); thus, conferring the stem cells with distinct levels of activity in terms of readiness to self-renew and fate decision.

Intestinal crypts are clonal units which are maintained by the continual division of the crypt stem cells. Before genetic manipulation and transgenic mice were available, ISCs were studied by analysis of somatic mutations. The rationale was that if the mutation occurs in the stem cell, the mutation will be fixed and the whole crypt will be composed of the mutated epithelium. The time which takes for the whole mutant crypt to appear i.e. the clonal stabilization time, has been the subject of studies in mouse and human intestine. Interestingly, differences have been observed in the cell kinetics of small vs. large intestine in mice. Campbell et al. investigated the mutation fixation in colectomy samples which had received radiotherapy prior to surgery. The proportion of somatic mutation fixation rose significantly one month after irradiation and reached the peak at 4-12 months. Subsequently, the proportion of partially mutated crypts decreased significantly with time i.e. at 4-12 months the majority of mutated stem cells have dominated the niche. Once the wholly mutated crypts appear, they persist in the colon for considerable length of time. This is suggestive of the total replacement of the stem cells by one or more ancestral mutated stem cell. Interestingly, the partially mutated crypts are not persistent. They either switch to wholly mutated crypts if the mutation is in the stem cell, or they will become normal again if the mutation is in the TACs (23). This is in accordance with a recent finding that heterozygous APC mutation will initiate intestinal tumourigenesis if it is induced in the stem cells and not TACs (24).

One caveat of mutation analysis is that it might not reflect the normal behaviour of ISCs (25). Yatabe et al. have examined methylation status of CpG islands of myogenic factor 3 (MYOD1), cardiac-specific homeobox (CSX) and an X-chromosome CpG-rich region in biglycan (BGN) to assess the epigenetic distance between crypts and indirectly study the stem cell dynamic in human colon. The results are presented in a binary system of 0 and 1; where 0 represents an unmethylated state whereas 1 is a methylated state. Epigenetic distance was defined as the absolute number of differences in methylation status of the studied genes; maximum of which was 5, 8, and 9 for MYOD1, CSX and BGN, respectively. The intracryptal and intercryptal epigenetic distances were calculated as the average epigenetic distance of all possible pairs of molecules within a crypt and between crypts, respectively. Applying genetic phylogenetic analysis, they argue that if there are multiple immortal stem cells in a crypt, the crypt will be polyclonal with each clone having a separate last common ancestor. However, if a stem cell dominates the niche, eventually the crypt will become monoclonal; which in phylogenetic terms equate to the origination of all cells from one last common ancestor. Interestingly, multiple unique epigenetic tags were identified in each crypt which suggests that crypts are not monoclonal (26).

Mathematical modelling and computational biology provide advantageous tools to assess biologic hypotheses which are not amenable to experimentation. Intestinal crypt homeostasis and intestinal tumorigenesis have been the topic of several mathematical models. Of relevance to the question of ISC heterogeneity, Lobachevsky and Radford have constructed a model based on the assumption that two tiers of stem cells exist in the intestinal crypt with distinct levels of activity and cycling patterns (27). This is in accordance with the experimental data in small intestine where CBC and position 4 cells appear to have non-overlapping functionality (4). However, experimental approach thus far has failed to produce the evidence for the existence of the slow-cycling stem cell in the colon. It is assumed that the cycling pattern of each stem cell type is the average of all its members. Therefore, it is conceivable that the stem cell pool is consistent of stem cells with distinct cycling rates. Hormoz suggests that stem cell heterogeneity in terms of the cell cycle rate results in an overall reduced rate of replicative aging in the tissue which is of importance in a rapidly proliferating tissue such as the intestine. It is further proposed that one single stem cell cycling rate is influenced by the microenvironmental signals. Nevertheless, the overall average is similar to that of the stem cell population (28). Monoclonal conversion of the intestinal crypt has been experimentally demonstrated in mouse duodenum. It was argued that niche succession occurs as a result of a neutral competition among stem cells which were assumed to be functionally homogeneous (29). Nevertheless, the possibility that some stem cells might outcompete their counterparts implies that the stem cell pool is concievably heterogeneous, at least transiently.

The hurdles of directly investigating ISC behavior were overcome by the availability of transgenic mice. Lineage-tracing experiments in mice have proven that a subset of cells highly expressing Lgr5, CD133, Bmi1, or mTert can repopulate the entire intestinal crypt (5-7, 30). It is noteworthy that in such experiments, only a subset of intestinal crypts is marked by the specific stem cell marker. In addition, distinct marking patterns have been observed in a lineage-tracing experiment where some crypts are marked continiously whereas others are either discontiniously marked or only a few marked cells form short v-like formations within a crypt (31). This is suggestive of different levels of activity of stem cells in different crypts i.e. intracryptal variation. Indeed, Lgr5^+^cells are found to be distinct in terms of proliferation activity as evidenced by the different levels of Ki67 expression. Based on the expression of Lgr5 and Ki67 four groups of cells were identified: Lgr5^high^Ki67^+^dividing ISCs, Lgr5^high^Ki67^- ^quiescent ISCs, Lgr5^low^Ki67^+^proliferating progenitors, and Lgr5^low^Ki67^-^quiescent progenitors (32). Niche succession i.e. dominating the entire crypt by the progenies of one stem cell is also suggestive of the intercryptal stem cell heterogeneity. Single cell imaging of hair follicle stem cell compartment revealed that stem cells have distinct behaviour depending on their position within the ‘niche’ in the hair follicle (33). It was previously shown that the proximity to Paneth cells determines the stemness of ISCs (34). Ritsma et al. have investigated spatial heterogeneity of Lgr5^+^ISCs at single cell level using multiphoton intravital microscopy through an abdominal imaging window. It was observed that stem cells rearrange following a division event which could eventually lead to removal of the border ISCs from the ‘niche’. In addition, it was observed that the position of the ISC within the crypt was a determining factor of its self-renewal capacity, i.e. cells located at positions 0 to+2 were more likely to retain their stemness whilst cells at positions+3 and+4 were more likely to divide into TACs (35).

Heterogeneity of ISCs can also be considered along the intestinal tract. Regional differences in crypt size, proliferative index and the distribution of proliferative cells along the crypt axis have been reported. For example, the crypt length is found to be significantly shorter in the ascending colon compared to transverse and descending colon. Moreover, the total number of proliferative cells is significantly lower in ascending colon compared to other colon segments. Other suggested variations include the cell cycle time, duration of mitosis, and apoptotic index post-irradiation (36, 37). Furthermore, the total number of Lgr5^+^stem cells significantly varies in different segments of the small intestine as well as between small intestine and colon in mouse and human. There is also a regional difference in the gene expression level of stem cell related genes and Wnt pathway modulators along the intestinal tract (38). In addition, not all crypts containes a non-Paneth label- retaining cell with only 10% having one and 0.1% having two such cells in the small intestine (39). Middendorp et al. have recently demonstrated that ISCs from different regions of the intestinal tract have distinct gene expression profile which is sustained following long *in vitro* organoid culture (40). Overall, it seems that stem cells vary along the intestinal tract.

Although, niche succession is a commonly accepted phenomenon in the intestinal crypt, the underlying mechanism is less well understood. The clock pathway is found to be required for ISC proliferative response following injury in Drosophila. Interestingly, clock-intact ISCs are found at different stages of the cell cycle, whereas ISCs are homogeneously either at G0 or S/ G2/ M phase irrespective of the time of the experiment upon RNAi clock knockdown (41). It is suggestive that the clock pathway maintains a degree of heterogeneity in the level of activity of ISCs. It was further shown that clock pathway regulates Wnt pathway components at gene expression level in the skin (22). This is not known in the intestinal epithelium and ISCs. Besides, the ISC hetero-geneity in terms of the clock pathway activity has not been investigated. However, the clock pathway is required for intestinal homeostasis (42). Although, the clock pathway has been shown to be functional in ISCs in Drosophila and was required for an appropriate proliferative response following dextran sodium sulphate treatment; it was not shown whether stem cells differ in the level of clock pathway activity (41). Other potential mechanisms which contribute to ISC heterogeneity need to be explored.

**Cancer stem cell heterogeneity**

It is long known that tumor tissue is composed of heterogeneous cell populations; a small fraction of which, i.e. cancer stem cells (CSC) have the capacity of self-renewal and sustaining the tumor evolution. Colorectal CSC are traditionally characterized by their ability to reproduce the original tumor hierarchy in mouse transplant experiments (43-45). However, the role of the microenvironment and an induced wound healing process are confounding factors in such experiments (46, 47). This issue is overcome in lineage tracing experiment. Murine experiments have clearly shown that tumorigenic hits can only result in an intestinal tumor if they happen in the stem cell compartment and not the transient-amplifying region (48). In a recent paper, Schepers et al. have demonstrated that APC-driven adenoma originates either from a single or multiple APC-mutated Lgr5^+^cells in mice (24). Moreover, constitutive activation of NF-κB and Wnt in intestinal epithelial cells results in the formation of aberrant crypt- like structures in the villi with high proliferation rate and high level of expression of stem cell markers. Interestingly, in the presence of constitutive NF-κB activity, a Wnt-activating mutation in the Lgr5^-^cell is sufficient to induce ‘stemness’ and initiate adenoma formation (49). If an ISC is affected by an oncogenic mutation, it could potentially result in the fixation of the mutation in a patch of cells which are histologically normal (field characterisation); providing that it retains its self- renewal capacity (50). Interestingly, ISC heterogeneity can be the result of an oncogenic mutation in the course of CRC tumorigenesis. For example, mosaic activated oncogenic K-ras^G12D^ in Lgr5^+^ISCs, results in growth advantage of mutated ISCs as evidenced by niche succession and clonal fixation by mutated ISCs (51).

It has recently been suggested that the CSC population is also heterogeneous with some cells more readily capable of tumor initiation and propagation than others (52). The CSCs heterogeneity has been demonstrated for breast and colorectal cancers (53, 54). Microenvironmental cues are suggested to fine-tune Wnt pathway activity in the course of intestinal tumorigenesis which in turn, can affect cancer stemness behavior of tumor cells. Despite the loss of APC in CRC, nuclear localization of β-catenin is not homogeneously observed in all tumor cells (55). Heterogeneous nuclear β-catenin staining is also observed in colonospheres. Gene expression comparison of colonosphere- derived cells with high vs. low Wnt pathway activity revealed that stem cell markers Lgr5 and Ascl2 are enriched in Wnt^high^ population whereas differentiation markers Muc2 and Krt20 are abundant in Wnt^low^ cells. Cells with high level of Wnt activity demonstrate higher level of clonogenicity and stemness features. Wnt^high^ colonosphere- derived cells are more efficient in reproducing the original tumor in immunocompromised mice (56). Colorectal cancer (CRC)-derived colonospheres were studied at single cell level. Each colonosphere was composed of cells with different proliferative capacity including postmitotic cells; cells which could undergo 1-3 divisions; and a fraction capable of self-renewal and sphere formation. The percentage of sphere forming cells varied in three tumor cells derived from primary tumors from different patients. Interestingly, the percentage of the sphere-forming cells remained stable in serial replating experiments for each patient. Using a cell tracking method in xenotransplantation experiment confirmed that tumors were polyclonal. Further investigation revealed that three populations of cells reproduced the tumor in mice: the so-called long-term tumor-initiating cell and tumor transient-amplifying cells; which were present in the primary tumour as well as following xenotransplants; and delayed-contributing tumor-initiating cells; which did not contribute to the first tumour formation in mice but appeared in the following rounds of serial transplantation (54). It is concluded that CRC-derived tumour-initiating cells are functionally heterogeneous.

Both normal intestinal and CRC cells demonstrate plasticity i.e. differentiated cells can dedifferentiate into cells with ‘stemness’ potential. Cellular heterogeneity within a tumor poses challenges regarding diagnosis, subtyping and treatment. Moreover, heterogeneity within CSCs adds another layer of complexity. However, unravelling genetic and microenviron-mental cues which regulate the level of ‘stemness’ of cells could conceivably be directly targeted in a therapeutic setting (57, 58).

**Conclusion and Perspective**

Lgr5^+^ISCs are often considered as collective homogeneous population of identical cells. However, different spatial positioning of the stem cells within a crypt plausibly results in a small yet effective difference in the level of the availability of stem cell regulatory molecules. This will in turn affect the transcriptomic landscape of each stem cell; which determines the stem cell behavior in terms of readiness to enter cell cycle as well as the choice between symmetric vs. asymmetric division. The fact that progenies of one stem cell dominate the crypt, suggests that some stem cells can outcompete others. In other words, stem cells are distinct in their capacity to populate the crypt. It is conceivable that a similar phenomenon to niche succession also exists in the course of CRC tumorigenesis where some CSCs are more active than others. The existence of colorectal CSCs with distinct tumor-initiating potential is supporting evidence to this hypothesis. CSC hypothesis proposes that cancer originates from a last common ancestral cell which is most likely the normal tissue stem cell. However, stem cell heterogeneity means that only a subset of stem cells can potentially be the cell-of-origin of the tumour. Therefore, it is crucial to discover the factors which underlie ISC heterogeneity to better understand the CRC tumorigenesis. Understanding the stem cell heterogeneity could then be a target for more selective cancer treatment.

## Conflict of interests

The author declared no conflict of interests.
